# Treatable traits in inflammatory bowel disease: a conceptual framework for personalized care

**DOI:** 10.1016/j.eclinm.2026.104058

**Published:** 2026-07-09

**Authors:** Carolina B. Graciolli Facanali, Celso R.F. Carvalho, Marcio Roberto Facanali, Carlos W. Sobrado, Charles N. Bernstein

**Affiliations:** a Department of Gastroenterology, Hospital das Clínicas, Faculdade de Medicina, Universidade de São Paulo, Brazil; b Department of Physical Therapy, Faculdade de Medicina, Universidade de São Paulo, São Paulo, Brazil; c University of Manitoba IBD Clinical and Research Centre, Winnipeg, Manitoba, Canada; d Department of Medicine, Max Rady College of Medicine, Rady Faculty of Health Sciences, Winnipeg, Manitoba, Canada

**Keywords:** Inflammatory bowel disease, Crohn's disease, Ulcerative colitis, Treatable traits, Precision medicine, Quality of life

## Abstract

Inflammatory bowel disease (IBD) is a complex, immune-mediated, and heterogeneous condition requiring individualized, mechanism-based care. Despite advances in therapies and treat-to-target strategies, achieving and maintaining sustained remission, as well as restoring health-related quality of life (HRQoL), remains challenging. The concept of *treatable traits* provides a framework for precision medicine grounded on identifying specific, measurable, and actionable mechanisms whose modification can improve outcomes meaningful to both patients and healthcare systems. This manuscript proposes a conceptual structure for applying the treatable-traits paradigm to IBD. It describes three primary domains: gastrointestinal and intrinsic, extraintestinal and comorbidity, and psychological and behavioral, integrating immunological mechanisms, systemic complications, and psychosocial determinants. The model emphasizes the bidirectional gut–brain axis and shared immune-inflammatory pathways linking intestinal inflammation with mental health. It also highlights how the STRIDE-II initiative and modern clinical trials have shifted therapeutic targets toward deep and sustained remission, explicitly including quality-of-life endpoints such as the IBD Quality of Life Questionnaire (IBDQ). Operationalizing treatable traits in clinical reasoning may bridge biological and “experiential remission”, helping clinicians prioritize actionable mechanisms, optimize therapy, and achieve the ultimate goals of IBD care: inflammation control, organ preservation, and restored wellbeing.

**Funding:**

None.

**Key messages**

• Treatable traits offer a multidimensional framework integrating intestinal, systemic, and psychosocial components of IBD.
• Implementing TT-based management could personalize care, enhance HRQoL, and reduce healthcare burden.
• Prospective TT-enriched studies are needed to validate clinical and cost-effectiveness across care levels.

## Introduction

Inflammatory bowel disease (IBD) encompasses chronic, relapsing immune-mediated disorders characterized by unpredictable flares of symptoms.^1^ IBD therefore represents not a single disease but a complex, multisystemic syndrome shaped by multifactorial interactions.^2^ The interaction between genetic susceptibility, environmental exposure, microbiota imbalance, and dysregulated mucosal immunity establishes a self-perpetuating inflammatory cycle that extends beyond the gut.^3^ Extraintestinal manifestations (EIMs) affect over 25% of patients, most commonly the joints, skin, and eyes, and contribute to a heterogeneous clinical course with a significant impact on health-related quality of life (HRQoL).

Despite therapeutic advances and progress from symptom control to treat-to-target paradigms aiming for mucosal healing and deep remission,^4^ only 40–50% of patients achieve sustained remission with current therapies.^5^^,^^6^ Commonly used outcome measures in clinical trials, such as the Mayo score or the Crohn's Disease Activity Index (CDAI), reflect global inflammation but neglect biological diversity and patient-reported outcomes. The *Selecting Therapeutic Targets in Inflammatory Bowel Disease* initiative (STRIDE-II) marked a conceptual turning point, redefining remission as comprehensive, encompassing clinical, biochemical, endoscopic, and patient-perceived wellbeing.^7^ Among the major innovations in the approach to IBD treatment, improvement in quality of life was recognized as an independent therapeutic target, reinforcing the notion that mucosal healing alone is insufficient to represent disease control.^8^

This convergence between biological remission and lived experience has been confirmed in pivotal trials of novel agents, anti-tumor necrosis factor (TNF), anti-integrin, anti-interleukin-23 (IL-23), janus kinase inhibitors (JAKi), and sphingosine-1-phosphate (S1P) modulators, which now include validated patient-reported outcome measures such as IBD Quality of Life Questionnaire (IBDQ), Patient Reported Outcome Measurement Information System (PROMIS), and Patient Reported Outcome-2 (PRO-2).^9^ These developments underscore the need for a framework that links objective inflammation suppression with subjective recovery: the *treatable-traits* model.

Yan et al. (2020) highlighted an apparent mismatch between physicians' clinical priorities and patients' expectations in IBD care; where physicians focus on disease activity, while patients are primarily concerned with improving overall wellbeing and HRQoL.^10^ Bridging these subjective clinical insights with the underlying objective biology is essential. HRQoL in IBD cannot be fully restored by mucosal healing alone; it depends on the resolution of systemic and neuroimmune disturbances that extend beyond the gut. It also depends on the affected individual's social interaction with their environment, including family, friends, workplace and/or school.

Expanding immunological understanding provides further coherence. The intestinal immune system operates as an integrated network of epithelial cells, innate lymphoid cells, macrophages, and T-cell subsets. Aberrant activation of *Th1* and *Th17* pathways, excessive signaling through IL-23, TNF-α, and IL-6, and disruption of epithelial integrity sustain chronic inflammation.^11^ These same cytokines circulate systemically, influencing metabolism and neural activity through the gut–brain axis. Pro-inflammatory mediators alter tryptophan–kynurenine metabolism and vagal signaling, leading to neuroinflammation, sleep disturbance, anxiety, and depression.^12^ Thus, inflammation, nutrition, and behavior represent interconnected mechanisms that are clinically observable and potentially modifiable.

**Fig. 1:**
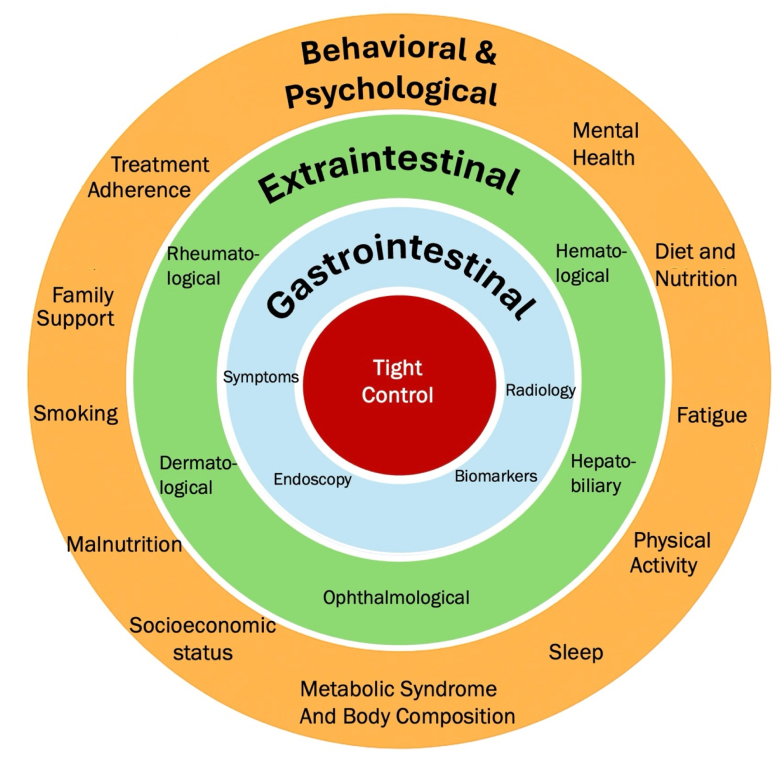
Conceptual framework of Treatable Traits in inflammatory bowel disease.

**Fig. 2:**
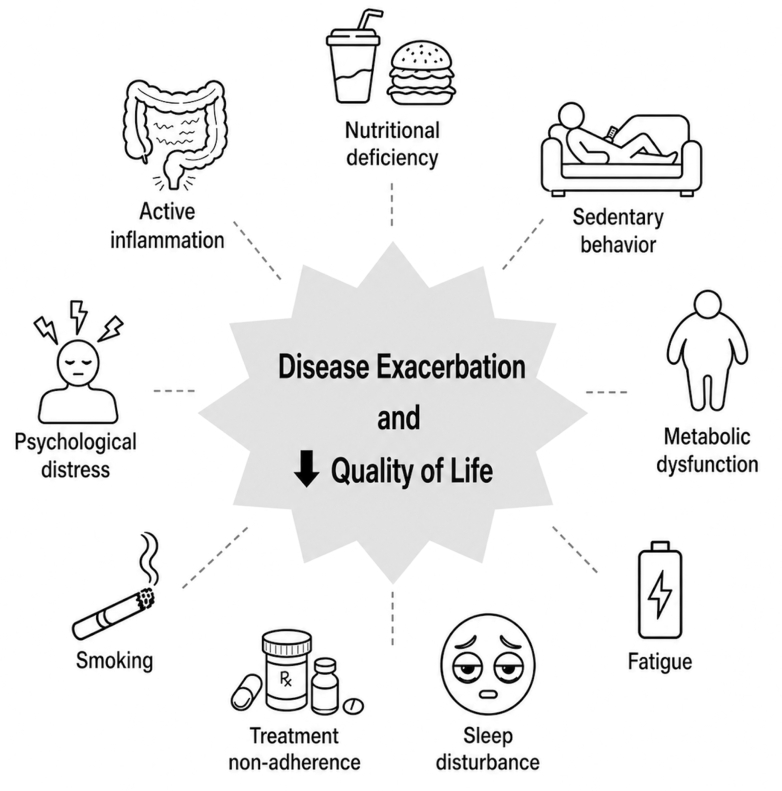
Interconnected drivers of disease exacerbation and impaired quality of life in inflammatory bowel disease. Inflammatory bowel disease (IBD) arises from the interaction of biological, metabolic, behavioral, and psychological factors that extend beyond intestinal inflammation. Clinically relevant and modifiable drivers, including active inflammation, nutritional deficiency, metabolic dysfunction, fatigue, sleep disturbance, sedentary behavior, smoking, treatment non-adherence, and psychological distress, contribute to disease exacerbation and impaired health-related quality of life (HRQoL), supporting an integrated, trait-based approach to care.

Recognizing all these interactions as *treatable traits* enables clinicians to move from static classification to a dynamic precision-care model grounded in mechanisms, measurability, and meaning. The treatable traits (TT) model, originally proposed for chronic airway diseases,^13^ categorizes clinically important, measurable, and modifiable factors linked to tailored interventions. Extending this concept to IBD could advance precision medicine, complement treat-to-target strategies, and improve patient-centered outcomes (Fig. 1). The clinical burden of IBD extends beyond inflammation alone, reflecting the interplay of multiple biological, metabolic, behavioral, and psychological drivers that contribute to disease exacerbation and impaired HRQoL (Fig. 2). The present study proposes a structured conceptual framework, aiming to link existing knowledge into a clinically applicable model.

## Framework development

The presented conceptual framework was developed through a structured narrative synthesis of existing evidence, integrating principles from treatable traits models in chronic diseases, established inflammatory bowel disease (IBD) management strategies, and emerging insights from precision medicine and biopsychosocial approaches, based on targeted most public accessible database searches using key topic-related terms relevant to the domains of interest, supplemented by relevant guideline documents and established references in the field. First, key conceptual foundations were identified from three major domains^1^: the treatable traits paradigm, originally described for chronic airway diseases,^13^ which emphasizes the identification of clinically relevant, measurable, and modifiable factors^2^; the STRIDE-II initiative,^7^ which defines a set of therapeutic targets in IBD; and^3^ contemporary models of gut–brain interaction and systemic inflammation.^12^ These frameworks were critically appraised and used as the theoretical backbone for domain selection and integration.

Second, domains and traits were designated based on three predefined criteria: clinical relevance, measurability in clinical settings, and potential for therapeutic modification. Traits were then grouped into three overarching domains (gastrointestinal and intrinsic; extraintestinal and comorbidity; and psychological and behavioral), reflecting the systemic nature of IBD and its systemic impact. Third, an integrative approach was adopted to organize these traits into a coherent, clinically interpretable structure. Each domain includes clinically relevant, measurable, and actionable mechanisms. The objective of this framework is to offer a conceptual and operational model that supports clinical reasoning and prioritization of modifiable mechanisms (Table 1). Finally, to enhance its clinical applicability, a stepwise workflow linking trait identification, stratification, prioritization, and targeted intervention was incorporated. This approach seeks to link the gap between conceptual models and real-world clinical decision-making, aligning biological disease control with patient-centered outcomes.

**Table 1:** Treatable Traits in Inflammatory Bowel Disease (IBD) grouped by domain, having the assessment tools, and conceptual therapeutic targets.

Domain	Treatable trait	Assessment	Target	Evidence maturity
Gastrointestinal and Intrinsic	Active inflammation (mucosal, transmural)	Fecal calprotectin, CRP, endoscopy (SES-CD), imaging (MRE, US), histology	Mucosal and transmural healing; cytokine suppression	Established
	Structural complications (strictures, fistulas)	Cross-sectional imaging, endoscopy, EUS	Prevent fibrosis; maintain luminal patency	Established
	Pharmacokinetic variability/immunogenicity	TDM (trough levels, anti-drug antibodies)	Therapeutic optimization; biologic persistence	Established
Extraintestinal and comorbidities	Anemia	Hb, ferritin, TSAT, CRP	Iron repletion; control of inflammatory activity	Established
	Osteoporosis	DEXA, vitamin D, calcium, corticosteroid history	Bone preservation; vitamin D supplementation	Established
	Primary sclerosing cholangitis, arthropathy, skin/eye manifestations	Clinical assessment, LFTs, MRI	Multidisciplinary management; systemic immune control	Established
Behavioral and Psychological	Diet and micronutrient status	Dietary recall, serum vitamins, B12, iron	Nutritional correction; dietary diversity; microbiota modulation	Emerging
	Sarcopenia/malnutrition	BMI, body composition (CT, BIA), handgrip strength, nutrition screening	Nutritional support; protein and energy balance	Established
	Smoking/tobacco exposure	Self-report, cotinine testing	Smoking cessation; relapse prevention	Established
	Fatigue	FACIT-F, IBD-F	Multidimensional fatigue management	Emerging
	Sleep quality	PSQI, actigraphy	Restorative sleep; circadian rhythm normalization	Emerging
	metabolic syndrome	BMI, waist circumference, imaging	Weight optimization; improve insulin sensitivity	Emerging
	Physical activity	IPAQ, accelerometer data	Regular moderate exercise; immune modulation	Emerging
	Family and social support	Qualitative interviews	Strengthened support networks; reduced stigma and isolation	Emerging
	Anxiety	GAD-7, HADS-A	Cognitive-behavioral therapy; mindfulness; stress reduction	Emerging
	Depression	PHQ-9, HADS-D	Psychotherapy ± pharmacotherapy	Emerging

BIA, bioelectrical impedance analysis; CRP, C-reactive protein; CT, computed tomography; DEXA, dual-energy X-ray absorptiometry; EUS, endoscopic ultrasound; FACIT-F, Functional Assessment of Chronic Illness Therapy–Fatigue; GAD-7, Generalized Anxiety Disorder 7-item scale; Hb, hemoglobin; HADS-A, Hospital Anxiety and Depression Scale–Anxiety subscale; HADS-D, Hospital Anxiety and Depression Scale–Depression subscale; IBD-F, Inflammatory Bowel Disease–Fatigue; IPAQ, International Physical Activity Questionnaire; LFTs, liver function tests; MRE, magnetic resonance enterography; MRI, magnetic resonance imaging; PHQ-9, Patient Health Questionnaire-9; PSC, primary sclerosing cholangitis; PSQI, Pittsburgh Sleep Quality Index; SES-CD, Simple Endoscopic Score for Crohn's Disease; TDM, therapeutic drug monitoring; TSAT, transferrin saturation; US, ultrasound.

The present framework does not intend to replace existing guidelines but to complement them by providing a structured approach for clinicians to interpret disease complexity and tailor personalized interventions. Future studies are required to validate its clinical utility and assess its impact on patient outcomes.

## Domains of treatable traits

### Gastrointestinal and intrinsic traits (from symptomatic treatment to deep remission)

The gastrointestinal and intrinsic domain represents the biological core of IBD. Mucosal inflammation results from dysregulated interactions among the intestinal epithelium, microbiota, and immune cells.^14^ Clinically, the inflammatory trait manifests as abnormal biomarkers, including fecal calprotectin (>150 μg/g), C-reactive protein (lab-dependent cutoff), anemia (serum hemoglobin <120 g/L in females and <130 g/L in males), or hypolbuminemia (<34 g/L). The therapeutic goal is to optimize pharmacotherapy, guided by pharmacokinetic monitoring in some cases and periodic biomarker evaluation to achieve a deep remission.^15^ When remission is achieved, mucosal and transmural healing is associated with reduced hospitalizations and improved HRQoL.^16^^,^^17^ Structural traits, including stricturing and penetrating patterns, represent chronic outcomes of uncontrolled inflammation. Distinguishing between inflammatory and fibrotic dominance directs treatment, from endoscopic dilation to combined medical-surgical strategies.

Beyond structural complications, chronic inflammation also contributes to an increased risk of colorectal cancer in patients with long-standing colonic IBD. The risk of colorectal cancer remains increased, and the cumulative inflammatory burden is influenced by disease duration and extent, being higher in extensive colitis. Risk is also increased in primary sclerosing cholangitis and in patients with a family history. Some extraintestinal diseases carry a heightened risk for cancer (e.g., primary sclerosing cholangitis and cholangiocarcinoma). In addition, some therapies carry a heightened risk for cancer (e.g., anti-TNF therapy and lymphoma and skin cancers), while there is an underlying heightened risk for specific cancers based on the sites of the underlying inflammatory disease (i.e., ileum small bowel adenocarcinoma; colon-colorectal cancer). According to ECCO guidelines, screening colonoscopy is recommended approximately eight years after symptom onset in patients with colonic involvement, followed by risk-stratified surveillance. Within a treatable-traits framework, malignancy risk integrates inflammatory control, dysplasia surveillance, and individualized risk assessment, which should be addressed within patient-centered care, given its impact on anxiety, patient concerns from the time of diagnosis, engagement, and as a persistent source of distress that may influence treatment adherence. Further, when inflammation is markedly reduced and endoscopic assessment suggests deep remission, surveillance intervals are lengthened to 5 years.^15^ Thus, intrinsic risks associated with the disease phenotype, the definition of its clinical scores, and disease activity are fundamental factors that contribute to identifiable traits.

### Extraintestinal and comorbidity traits (the known extraintestinal manifestations and associated comorbidities)

Systemic involvement reflects the extraintestinal propagation of inflammation and metabolic disturbance.^18^ Circulating cytokines influence hematopoiesis, bone remodeling, and muscle catabolism, generating a cluster of treatable systemic traits. Among the extraintestinal traits are anemia and osteopenia. Anemia, present in up to 45% of patients, arises from iron deficiency and chronic inflammation.^19^ Correction through iron replacement and inflammation control improves fatigue, cognition, and productivity.

Osteopenia and osteoporosis can arise from corticosteroid exposure and from the interference of pro-inflammatory cytokines with osteoblast differentiation^20^ Preventive screening and supplementation to protect bone health are good examples of the management of systemic traits. Immune-mediated extraintestinal manifestations, including arthropathy, erythema nodosum, pyoderma gangrenosum, iritis, episcleritis, and primary sclerosing cholangitis, share immunologic roots with intestinal inflammation via lymphocyte trafficking and antigen cross-reactivity. Multidisciplinary recognition of these traits aligns with holistic remission goals defined by STRIDE-II.

### Behavioral and psychological traits (from lifestyle and behaviors to mental health)

Psychological and behavioral traits are central to understanding IBD as a systemic disorder with intertwined neuroimmune and psychosocial dimensions.^21^ They represent biologically grounded, modifiable mechanisms that influence inflammation, coping, and HRQOL. At least nine key traits illustrate the bidirectional interplay among the brain, gut, and immunity. They include diet, smoking, fatigue, sleep quality, metabolic syndrome and body composition, physical activity, treatment adherence, family and social support, and mental health.

#### Diet and nutrition

Dietary patterns and micronutrient status represent key modifiable traits in IBD.^22^ Nutrition has a direct influence on mucosal immunity, microbial diversity, and the gut–brain axis.^23^ Deficiencies in iron, vitamin D, and vitamin B12 contribute to fatigue, cognitive impairment, and bone loss. Rather than prescribing restrictive diets, the treatable-traits approach encourages individualized assessment and correction of deficiencies, aligning dietary guidance with the control of inflammation and emotional wellbeing. Malnutrition and sarcopenia, promoted by inflammatory catabolism and corticosteroid use, predict surgical complications and impaired HRQOL.^24^^,^^25^ There has been increasing interest in modifying diets to include more high-fiber foods and minimize consumption of ultraprocessed foods, which, in turn, may positively affect the gut microbiome.^26^ Other diets are being considered to address symptoms that may be unrelated to inflammation, such as the low fermentable oligo-, di-, mono-saccharides and polyols (FODMAP) diet, early nutritional screening, and rehabilitation that address these traits directly.^27^^,^^28^ Diet involves eating patterns, emotional regulation, and lifestyle choices and is behavioral,^29^ but could also be considered in relation to gastrointestinal traits since some aspects of diet may be driven by intestinal inflammation and structural changes.

#### Smoking (tobacco use)

Smoking status remains a critical behavioral trait influencing disease phenotype and outcomes, at least in Western populations, especially in Crohn's disease.^30^ Tobacco exposure aggravates inflammation, accelerates postoperative recurrence, and reduces therapeutic response. Smoking cessation represents a cornerstone treatable trait that should be systematically addressed in every patient, integrated with psychological and educational support.

#### Fatigue

Fatigue is one of the most prevalent and disabling traits in IBD, reflecting the convergence of inflammation, anemia, sleep disruption, emotional distress, and possibly circadian rhythm disruption.^31^ Addressing fatigue conceptually involves evaluating it as a construct encompassing physical, cognitive, and emotional, requiring integrated management across both biological and psychological domains. Recently, in a large cohort study of persons with IBD, anxiety, depression, and active gastrointestinal symptoms were all significantly associated with reporting high fatigue.^32^

#### Sleep

Sleep disturbances are highly prevalent in IBD, even during clinical remission.^33^ Fragmented sleep alters circadian cortisol rhythms, increases IL-6 and TNF-α production, and disrupts epithelial barrier integrity. Patients with poor sleep show higher fecal calprotectin and relapse risk.^34^ Sleep is closely interconnected with physical activity and mental health, forming a regulatory triad for neuroimmune homeostasis. Adequate sleep supports neuroendocrine balance, emotional stability, and improved physical recovery, while persistent insomnia fuels fatigue and psychological distress.^35^ Recognizing sleep as a central treatable trait reinforces its role as a mediator between the gut–brain axis and immune regulation.

#### Metabolic syndrome and body composition

In the context of IBD, metabolic syndrome, encompassing insulin resistance, dyslipidemia, hypertension, and visceral adiposity, is critical as it may interact with chronic inflammation and influence disease outcomes. Within this context, obesity represents a heterogeneous construct, with distinct phenotypes, including visceral adiposity, sarcopenic obesity, and metabolically unhealthy normal-weight individuals, who may carry an increased and often underrecognized cardiovascular risk, with distinct clinical and prognostic implications. Lean individuals may also suffer from metabolic syndrome or components thereof (i.e., hypertension or diabetes), and so these conditions need to be considered in all patients regardless of BMI.

Adipose tissue acts as an active immunological organ, amplifying systemic inflammation and modifying drug pharmacokinetics.^36^ Excess visceral fat and sarcopenic obesity correlate with more severe disease, postoperative complications, and reduced response to biologic therapies. Trait-oriented management integrates gradual weight optimization with physical activity and nutritional rebalancing to restore inflammatory homeostasis and overall function.

On the other hand, being underweight with a low BMI may also reflect psychological issues ranging from anorexia nervosa to avoidant restrictive food intake disorder to gastrointestinal inflammation and/or fibrosis that limits or modifies oral intake. Management of this trait extends beyond dietary interventions and includes behavioral strategies, pharmacological therapies, and, in selected cases, endoscopic or surgical approaches, underscoring the need for a comprehensive individualized approach to the management of metabolic and body-composition-related abnormalities in IBD.

#### Physical activity

Although exercise and physical activity are not the same concept, the World Health Organization recommends physical activity for the general population. Recently, studies have shown that regular physical activity improves HRQoL, parent-reported fatigue, and clinical disease activity.^37^^,^^38^ Enhancing physical activity and exercise thus emerges as a pivotal *treatable trait*, integrating metabolic, immune, and psychological pathways and acting as a bridge between body and mind. Physical inactivity is both a consequence and an amplifier of chronic inflammation^39^; it may play a causal role in the development of many gastrointestinal disease.^40^^,^^41^ Additionally, exercise exerts broad immunomodulatory effects upregulating IL-10 and IL-1ra, while reducing CRP, IL-6, and TNF-α.^42^^,^^43^ A recent study showed that low socioeconomic status is a key driver of low physical activity in a large cohort of persons with IBD.^32^

#### Treatment adherence

Some patients commonly have difficulty following medical advice, medication regimens, diets, or lifestyle changes. Treatment adherence may be related to the relationship with healthcare professionals or to patient-specific factors. Around one-third of patients adequately adhere to treatment,^44^ and the lack of adherence is probably the most important cause of therapeutic failure in people with chronic diseases^40^ The prevalence of non-adherence to drug treatment in Crohn's disease ranges from 30% to 63%.^45^ Among the important consequences of poor adherence are a substantial increase in the risk of clinical relapse, colorectal carcinoma, and disability.^46^^,^^47^ Why do some patients resist following medical recommendations? Doubts about the effects of medications and concerns about possible side effects have been reported as the root of non-adherence.^48^ Many patients have anxiety and mood disorders, and it is evident that some refuse therapy due to indifference toward the diagnosis or show no interest in receiving treatment.^49^^,^^50^ Other factors, such as a negative perception of treatment, were identified as the main cause of non-adherence among IBD patients in Turkey.^51^ In India, therapy costs, absence of symptoms (presumed cure), and fear of adverse effects were reported as reasons for non-adherence.^52^

Adherence to IBD treatment is, therefore, a complex and multifactorial issue, yet essential for maintaining long-term remission and preventing complications. Patient education is also incorporated as a relevant and modifiable component within this domain. Beyond a physician-centered perspective, educating patients and their families can improve treatment adherence, lifestyle behaviors, psychological wellbeing, and engagement with care by strengthening health literacy and facilitating shared decision-making. In this context, placing the patient at the center of care and promoting shared responsibility for disease management may enhance long-term adherence and self-management. Patient empowerment and advocacy may not only improve individual outcomes but also increase disease awareness and promote more equitable healthcare systems.

#### Family and social support

The psychological suffering of an individual upon receiving the diagnosis of IBD may be substantial, and a lack of support is one of the primary causes negatively affecting HRQoL, mental health, and social relationships.^53^ The natural course of the disease demands prolonged treatment and impairs the ability to meet basic daily needs such as nutrition, hygiene, and safety. Social status, in turn, impacts the disease course. The outcomes for persons with IBD who are of lower socioeconomic status are significantly worse. The severe physical impact that IBD may impose on patients’ health leads to significant limitations in everyday life. Even during remission, the heightened concerns brought by the disease carry major consequences for psychological and social wellbeing, reducing HRQoL. A Canadian study concluded that IBD significantly affects the whole family in terms of interpersonal relationships, HRQoL, and everyday activities such as diet, travel, finances, family planning, and leisure.^54^

A recent systematic review of the impact of IBD on family members also showed strong influence on emotional wellbeing, social life, work, finances, travel, and leisure^55^ Family and social support modulate stress perception and treatment response.^56^ Supportive networks correlate with lower anxiety, greater adherence, and higher satisfaction with care, whereas isolation and stigma exacerbate fatigue and depression. Family counseling, patient groups, and peer mentoring strengthen resilience and empowerment. Social support, therefore, constitutes a critical and treatable determinant of prognosis and quality of life.

#### Mental health

Anxiety affects approximately one-third of patients and correlates with inflammatory activity and poorer self-perceived health.^57^ Cytokines such as IL-6 and TNF-α modulate neurotransmission and hypothalamic–pituitary–adrenal axis reactivity can link the immune activation to anxious behavior. Persistent anxiety heightens visceral sensitivity and impairs adherence. Screening with validated instruments (e.g., GAD-7, HADS-A) may help to identify mental health needs, and interventions such as cognitive-behavioral therapy or mindfulness may help reduce symptoms and restore gut–brain equilibrium. Anxiety thus represents a biologically anchored, modifiable trait bridging immunity and emotion.

Depression shares overlapping immunologic and neurochemical pathways with intestinal inflammation. Elevated IL-1β, IL-6, and TNF-α levels and altered tryptophan metabolism correlate with depressive symptoms and predict worse disease outcomes.^58^^,^^59^ Facanali et al. emphasized that depression in IBD is not merely a psychological reaction to chronic illness but may reflect underlying neuroimmune dysregulation, representing a critical target for comprehensive and personalized intervention.^60^ Integrated psychotherapeutic and pharmacologic strategies can optimize immune response and promote sustained remission. Depression, therefore, constitutes a treatable trait with clear physiological relevance.

## Clinical implications and conceptual integration

The value of the treatable-traits paradigm lies not in prescriptive algorithms but in providing a conceptual language for a structured precision care approach. It reframes how clinicians and researchers interpret mechanisms, therapeutic goals, and the meaning of remission. Conceptually, the framework unites biological precision with experiential precision. Identifying traits transforms complex pathophysiology into observable, interconnected phenomena: inflammation, sleep, fatigue, and mood cease to be isolated events and become part of an interconnected clinical continuum.

This approach expands the logic of STRIDE-II, grounding its multidimensional goals, clinical, endoscopic, biochemical, and HRQOL, within a single interpretive structure. While STRIDE-II defines comprehensive therapeutic targets in IBD, it does not explicitly provide a structured approach for prioritizing and integrating these targets in individual patients. In this context, the treatable traits framework offers a complementary model, providing a structured approach to prioritize and operationalize these targets at the individual level. The treatable-traits model does not replace traditional reasoning; it deepens it by helping clinicians prioritize what is most modifiable and meaningful to each patient at a given moment.

It is important to recognize that not all treatable traits are supported by the same level of evidence. Some traits, such as active inflammation, anemia, and structural complications, are well-established and supported by robust clinical and mechanistic data. In contrast, other traits, particularly within the behavioral and psychological domains (e.g., sleep quality, fatigue, treatment adherence, and social support), represent emerging or evolving areas of research, where evidence is growing but remains heterogeneous. Distinguishing between established and emerging traits may help guide clinical prioritization and inform future research directions.

**Fig. 3:**
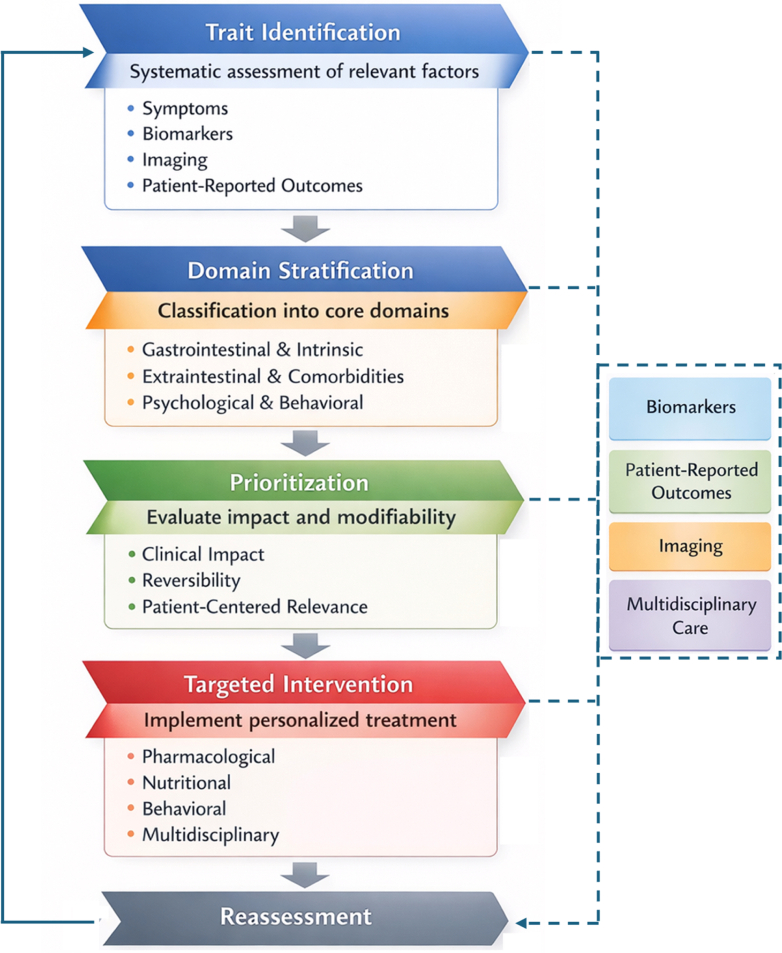
Clinical workflow for the implementation of treatable traits in inflammatory bowel disease.

In research, trait-based inclusion criteria could replace rigid diagnostic segmentation. Future trials may stratify participants by predominant mechanisms such as inflammatory, metabolic, or psychosocial, to yield more targeted and translatable results. Ultimately, integrating the gut–brain–immune axis into IBD management reframes mental health as a core therapeutic frontier rather than a supportive adjunct. Achieving deep and sustained remission will depend on simultaneously addressing cytokines, behaviors, and context. While the treatable traits framework provides a conceptual foundation for comprehensive care, its clinical utility depends on its translation into structured decision-making processes. To enhance the clinical applicability of the treatable traits framework, we propose a structured, stepwise workflow that translates conceptual domains into practical decision-making (Fig. 3).

First, clinicians perform systematic trait identification, integrating clinical evaluation, biomarkers, imaging, and patient-reported outcomes to capture both biological and experiential dimensions of disease. Second, identified traits are stratified across domains (gastrointestinal and intrinsic, extraintestinal and comorbidity, and psychological and behavioral), allowing a structured characterization of disease burden. Third, traits are prioritized based on clinical impact, reversibility, and patient-centered relevance, recognizing that not all traits require simultaneous intervention. This prioritization is essential to avoid therapeutic overload and to align treatment with the most meaningful drivers of disease activity and impaired quality of life. Fourth, clinicians implement targeted interventions, including pharmacological optimization, nutritional strategies, behavioral interventions, or multidisciplinary care, depending on the dominant traits. Finally, the model emphasizes dynamic reassessment, where traits are periodically re-evaluated to guide treatment adaptation, reflecting the evolving nature of inflammatory bowel disease.

The proposed workflow provides a pragmatic framework for operationalizing the treatable traits concept in routine practice, bridging the gap between complex disease understanding and individualized care. By integrating conceptual understanding with a practical workflow, this model supports a more structured and individualized approach to IBD management in real-world settings.

## Future directions

The treatable-traits paradigm offers an integrated research and clinical framework that unites the biological, psychological, and social sciences within a patient-centered model. Its application in IBD is emerging but expanding rapidly. Standardized definitions and validated tools are urgently needed, particularly for subjective traits such as sleep, fatigue, adherence, and social support. Consensus frameworks analogous to STRIDE-II targets would enable reproducibility and multicenter comparability.^7^

Future longitudinal and interventional studies should determine the extent to which modifying specific traits improves clinical, functional, and psychosocial outcomes. Multidomain assessments may uncover cross-domain effects, for instance, whether addressing psychological distress influences inflammatory biomarkers, thus confirming mechanistic coherence.^61^ Integrating multi-omics and digital phenotyping could further refine trait identification, linking molecular profiles with behavioral data. Artificial intelligence may model these interactions, enhancing relapse prediction and individualized care.

Education and implementation science are pivotal for translating this approach into practice. Training clinicians to reason in mechanisms rather than diagnostic categories, supported by digital tools that summarize active domains, may accelerate adoption. Ultimately, aligning therapeutic priorities with patients’ perspectives ensures that success reflects both biological control and lived experience. Embedding treatable traits into IBD care thus represents a path toward more personalized, mechanistically driven, and patient-empowered disease management.

The proposed framework has some limitations, as it is conceptual and does not include primary empirical data or prospective validation. Although it is grounded in existing evidence and expert-informed integration, its clinical utility and impact on patient outcomes require validation in future observational and interventional studies. Additionally, the absence of standardized criteria for trait selection and prioritization highlights the need for consensus development and methodological refinement.

## Conclusions

The treatable-traits framework redefines IBD management as the dynamic interaction of biological, systemic, and psychological mechanisms rather than static diagnostic entities. By identifying and targeting modifiable factors across these domains, clinicians can move toward a more integrated and humanized precision medicine. The convergence of immune, neuroendocrine, and behavioral pathways supports the inclusion of mental health, sleep, physical activity, adherence, and social support as measurable therapeutic targets.

This multidimensional approach complements STRIDE-II by operationalizing its clinical, endoscopic, and HRQoL goals within a unified logic that expands remission to include functionality and emotional wellbeing. Rather than replacing current guidelines, the treatable-traits paradigm enriches them by providing a structured method to prioritize patient-specific modifiable domains. Aligning precision medicine with person-centered care thus represents the next step in IBD management, where success is measured not only by inflammation control but by restoration of full health and HRQoL.

## Outstanding questions

• Can treatable trait-based strategies improve inflammatory outcomes in inflammatory bowel disease?
• Which treatable traits should be prioritized according to inflammatory bowel disease phenotype and disease activity?
• How should treatable traits be longitudinally monitored and integrated into tight-control strategies?
• Could a treatable-traits framework advance precision medicine and interdisciplinary care in inflammatory bowel disease?

## Contributors

Conceptualization: CBGF, CRFC, CWSJ, CNB.

Study design: CBGF, CRFC, MRFJ, CWSJ, CNB.

Writing—original draft: CBGF.

Writing—review & editing: CBGF, CRFC, MRFJ, CWSJ, CNB.

Final approval of the manuscript: CBGF, CRFC, MRFJ, CWSJ, CNB.

CBGF and CRFC accessed and verified the underlying data. Authors were not precluded from accessing the study data, and they accept responsibility for the decision to submit for publication.

## Declaration of interests

**CNB** is supported by the Bingham Chair in Gastroenterology. Dr. Bernstein has served on advisory boards for AbbVie Canada, Amgen Canada, Bristol Myers Squibb Canada, Celltrion, Eli Lilly Canada, Ferring Canada, Fresenius-Kabi Canada, Janssen Canada, Pendopharm Canada, Sandoz Canada, Takeda Canada, and Pfizer Canada; Educational grants from AbbVie Canada, Boston Scientific, Bristol Myers Squibb Canada, Eli Lilly, Ferring Canada, Pfizer Canada, Takeda Canada, Janssen Canada, Organon Canada, Eli Lilly Canada, and Amgen Canada. Speaker's panel for AbbVie Canada, Fresenius-Kabi Canada, Janssen Canada, Pfizer Canada, and Takeda Canada. Received research funding from AbbVie Canada, Amgen Canada, Sandoz Canada, Takeda Canada, and Pfizer Canada.

**CBGF** has served on advisory boards for Johnson & Johnson; has participated in speaker panels and educational activities for Johnson & Johnson, Pfizer, and Takeda; has received educational grants from AbbVie, Johnson & Johnson, Nestlé, Pfizer, and Takeda; has received travel support from AbbVie, Johnson & Johnson, Pfizer, Takeda, Nestlé, and ABCD (Associação Brasileira de Colite Ulcerativa e Doença de Crohn); and has been involved in the development of educational materials for Johnson & Johnson and Takeda. CBGF also reports scientific and professional involvement with GEDIIB (Organização Brasileira de Doença de Crohn e Colite) as a member of the Surgical Committee of GEDIIB (unpaid, 2025–2026).

**CWSJ** reports speaker panels for AbbVie, Johnson & Johnson, Takeda, and FQM, and educational grants from Johnson & Johnson.

**CRFC** has a personal research scholarship funded by the Conselho Nacional de Pesquisa de Desenvolvimento Científico e Tecnológico (CNPq) (grant 312279/2018-3).

**MRFJ** reports no conflicts of interest.
